# Apolipoprotein A1 reduces blood–spinal cord barrier leakage, improves astrocytic coverage, and enhances motor neuron survival to restore the neurovascular unit in ALS mice

**DOI:** 10.3389/fnagi.2025.1684694

**Published:** 2026-01-12

**Authors:** Svitlana Garbuzova-Davis, Larai Manora, Cesario V. Borlongan

**Affiliations:** 1 Center of Excellence for Aging and Brain Repair, Morsani College of Medicine, University of South Florida, Tampa, FL, United States; 2 Department of Neurosurgery, Brain and Spine, Morsani College of Medicine, University of South Florida, Tampa, FL, United States

**Keywords:** ALS, apolipoprotein A1, G93A SOD1 mutant mice, mouse sex, behavior, blood–spinal cord barrier permeability, astrocytes, microglia

## Abstract

**Introduction:**

Amyotrophic lateral sclerosis (ALS) is a progressive, age-related motor neuron degenerative disease with multiple causal factors. Dyslipidemia has been identified as an important pathological element. Impaired lipid protein metabolism manifests in ALS patients and in an ALS mouse model. Apolipoprotein components are the primary regulators of plasma lipid metabolism. Apolipoprotein A1 (ApoA1), a high-density lipoprotein, acts as an antioxidant and reduces inflammation, preventing blood vessel injury. However, the effects of ApoA1 upon the ALS-damaged endothelium in the CNS are unknown. The objective of the study was to determine the effect(s) of injecting ApoA1 into G93A SOD1 mice at the early symptomatic stage.

**Methods:**

A single dose of ApoA1 or media was systemically administered into 13-week-old G93A SOD1 male and female mice. Body weight and tests of motor function were evaluated weekly for 4 weeks post-injection. Permeability of spinal cord capillaries was determined by Evans blue (EB) fluorescent dye injected into mice at 17 weeks of age. Immunohistochemical analyses determined the statuses of glial cells and ApoA1 distributions in ALS mice cervical/lumbar spinal cords. Motor neurons in cervical/lumbar spinal cord ventral horns of ApoA1-treated and media-injected ALS mice were stained with cresyl violet for histological analyses.

**Results:**

ApoA1 injected into G93A SOD1 mice at the early symptomatic stage significantly benefited both male and female animals by (1) delaying behavioral disease progression; (2) reducing EB capillary leakage into spinal cord parenchyma; (3) lessening astrogliosis and microgliosis; (4) protein incorporation into capillary endothelium and motor neurons; and (5) improving survival of motor neurons in the spinal cord.

**Conclusion:**

Our novel data showed that systemically administered ApoA1 benefited ALS mice of both sexes, likely by beneficial effects on damaged microvessels, possibly engendering restoration of neurovascular unit integrity. Moreover, an anti-inflammatory ApoA1 effect was demonstrated by the reduction of glial cell activation, potentially mitigating vascular injury. The results of our preclinical study suggest that ApoA1 may be a potential protein-mediated therapeutic for restoring vascular function. Our novel strategy may lead to future clinical trials, furthering our goal of effectively treating ALS patients.

## 1 Introduction

Amyotrophic lateral sclerosis (ALS) is a fatal age-related disease of widespread motor neuron degeneration in the brain and spinal cord, affecting both males and females (McCombe and Henderson, 2010; Mehta et al., 2022). Treatment for ALS is mainly palliative. Among multiple factors involved in disease pathogenesis, dyslipidemia is considered an essential component of the pathologic disease process (Joardar et al., 2017; González De Aguilar, 2019; Chełstowska et al., 2021). Dysregulation of lipoprotein metabolism was noted in ALS patients (Mariosa et al., 2017; Chaves-Filho et al., 2019; González De Aguilar, 2019; Xia et al., 2023) and a mouse ALS model (Fernández-Beltrán et al., 2021). Higher blood levels of lipids such as high-density lipoprotein (HDL), low-density lipoprotein (LDL), apolipoprotein A1 (ApoA1), and apolipoprotein B (ApoB) correlated with an increased risk of ALS. Mainly, a gradual decline in ratios between LDL and HDL and/or ApoB and ApoA1 was observed in ALS patients. A recent study (Chalitsios et al., 2024) provided evidence that LDL and total cholesterol are implicated in ALS, and an increased ApoB level is related to ALS and frontotemporal dementia (FTD). The authors suggested that “ApoB inhibition might decrease the risk of sporadic ALS and FTD” (Chalitsios et al., 2024).

Lipoprotein metabolism is mainly regulated by various apolipoproteins in plasma by their involvement in the transport of lipids among cells and tissues (Koschinsky and Marcovina, 2004).

Specifically, ApoB, an LDL component, is involved in the development of cardiovascular diseases (Benn, 2009; Morita, 2016). In contrast, ApoA1, a major HDL protein, provides anti-oxidative, anti-inflammatory, and anti-thrombotic actions (Kontush et al., 2003; Mineo et al., 2006; Walldius, 2012; van der Vorst, 2020; Tao et al., 2024). This protein is essential for cholesterol transport and cholesterol balance in cells, preventing vascular damage. Elevated ApoA1 levels are associated with decreased risk of coronary diseases, while high ApoB levels and high ApoB/ApoA1 ratios were related to increased cardiovascular events (Walldius et al., 2004; Walldius and Jungner, 2006). An imbalance between ApoB and ApoA1 may be an etiological mechanism for ALS (Mariosa et al., 2017), and increasing HDL/ApoA1 in blood might reduce the development of ALS (Thompson et al., 2022). In general, higher levels of HDL, specifically ApoA1, and lower LDL/HDL ratios are associated with reduced risk of ALS.

ApoA1 has shown beneficial effects in the treatment of atherosclerosis, thrombosis, diabetes, and other cardiovascular-related injuries (Smith, 2010; Wang et al., 2010; Cochran et al., 2021) via cholesterol reversal transport (Bhale and Venkataraman, 2022; Xu et al., 2022). In addition, reparative processes of ApoA1 were determined by regulating immune cell functions (reviewed in Tao et al., 2024). For example, ApoA1 has been observed to reduce neutrophil counts (Kingwell et al., 2024) and inhibit antigen-presenting ability of monocytes in patients with acute myocardial infarction (Richart et al., 2020), exerting anti-inflammatory effects by modulation of immune cell interaction. Furthermore, numerous studies provided evidence of anti-inflammatory ApoA1 actions on inhibition of endothelial cell (EC) and arterial inflammation (Shah et al., 1998; Yan et al., 2006; Li et al., 2008; Puranik et al., 2008; Pan et al., 2016; Svensson et al., 2017). Interestingly, ApoA1 treatment resulted in a reduction of inflammatory EC response and promoted vascular repair in a mouse model of type 2 diabetes (Babu et al., 2023). Molecular mechanisms of ApoA1 actions on reducing EC inflammation revealed increased annexin A1 and inhibition of the activation of phospholipase A_2_ (Pan et al., 2016). Moreover, ApoA1 up-regulated angiopoietin 4 in human ECs via the phosphoinoside 3-kinase (PI3K)/Akt/FOX01 downstream signaling pathway (Theofilatos et al., 2018). Blockage of the PI3K/Akt-dependent signaling pathway with wortmannin has also been shown to prevent microvesicle-induced angiogenesis, likely due to inhibiting the transfer of proteins to ECs (Deregibus et al., 2007). Thus, in addition to maintaining cellular cholesterol homeostasis, ApoA1 offers anti-inflammatory effects through the regulation of vascular ECs.

We (Garbuzova-Davis et al., 2007a,b, 2012) and others (Zhong et al., 2008; Henkel et al., 2009; Nicaise et al., 2009; Miyazaki et al., 2011; Winkler et al., 2013) demonstrated impairment of the blood–spinal cord barrier (BSCB) in ALS animal models and in ALS patients, mainly by capillary EC alterations. Since BSCB damage represents an additional element in ALS pathogenesis, ALS might be classified as a neurovascular disease (Garbuzova-Davis et al., 2011). Repair of the impaired barrier is essential to prevent various detrimental agents in systemic circulation from entering the CNS, which could exacerbate ALS motor neuron degeneration.

Although ApoA1 has shown a promising role in maintaining ECs, it is necessary to determine ApoA1’s effects on the disease-damaged CNS endothelium and other cellular constituents in ALS. Our relatively recent study (Garbuzova-Davis et al., 2022) demonstrated the therapeutic potential of ApoA1 for endothelial cell repair under pathologic conditions reminiscent of ALS. Study results showed significantly increased mouse brain EC death upon exposure to plasma from G93A SOD1 mice with initial disease symptoms *in vitro*. Adding ApoA1 to cultured ECs substantially reduced cell death in a dose-dependent manner via protein incorporation into ECs. Since our *in vitro* study identified ApoA1 as a potential therapeutic, further research was needed to determine the *in vivo* effects of ApoA1 in restoring the ALS-damaged endothelium. Moreover, cellular characteristics, such as perivascular astrocytes and motor neurons, are essential to provide evidence whether protein-facilitated CNS homeostasis is an effective treatment for ALS. In this context, ApoA1’s potential integration into motor neurons is important to establish for proper cell maintenance.

The aim of this study was to determine the effects of ApoA1 administered into male and female G93A SOD1 mutant mice at the early symptomatic disease stage.

## 2 Materials and methods

### 2.1 Ethics statement

Detailed experimental approaches were approved by the Institutional Animal Care and Use Committee at USF (IACUC # R IS00011795) and conducted in accordance with the *Guide for the Care and Use of Laboratory Animals*.

### 2.2 Animals

Mice for the study were received from The Jackson Laboratory, Bar Harbor, MA, USA. In total, 43 transgenic male and female B6SJL-Tg(SOD1*G93A)1Gur/J mice, RRID:IMSR_JAX:002726, overexpressing human SOD1 possessing the Gly93→Ala mutation (G93A SOD1) at 7 weeks of age, were randomly assigned to one of two groups receiving ApoA1 or media: *Group 1*—ApoA1 (100 μg/mouse, *n* = 11/sex) and *Group 2*—Media (males *n* = 10; females *n* = 11). Beginning weekly at 8 weeks of age, pretreatment mice experienced behavioral testing (grip strength, rotarod, and extension reflex) and body weight monitoring. Upon initial disease symptoms at approximately 13 weeks of age, such as deteriorating motor function and reduced body weight, mice received an intravenous injection (200 μL, jugular vein) of either ApoA1 solution or media. A non-transplant control group (*Group 3*), consisting of non-carrier mutant SOD1 gene mice from the background strain (control, *n* = 9/sex), only underwent behavioral testing. Weekly from 14 weeks until 17 weeks of age, all mice were again tested for behavioral outcomes.

The G93A SOD1 mouse model of ALS, developed by Gurney et al. (1994), provides an excellent tool for basic research of ALS pathogenesis. This transgenic animal model carries a mutation in the human SOD1 gene, leading to progressive motor neuron degeneration that resembles human ALS. We (Garbuzova-Davis et al., 2017, 2018, 2019, 2021) and other researchers (Zhong et al., 2008; Nicaise et al., 2009; Miyazaki et al., 2011; Winkler et al., 2013; Fernández-Beltrán et al., 2021) widely used the G93A mouse model for molecular and/or cellular mechanisms of disease and extensively studied the efficacy of potential therapies for ALS. Initiating ApoA1 treatment at early symptomatic ALS mice in the current study mimicked the clinical presentation of ALS patients, and this choice was supported by our previous publications (Garbuzova-Davis et al., 2018, 2019). The ApoA1 (100 μg) matched a dose from our *in vitro* study (Garbuzova-Davis et al., 2022) that demonstrated therapeutic benefits of ApoA1 for endothelial cell repair.

### 2.3 ApoA1 preparation and administration procedure

ApoA1 from human plasma with low endotoxin levels (Athens Research & Technology, Athens, GA, USA, Cat. No. 16-16-120101-LEL) was used. According to the company report, ApoA1 obtained from human plasma was demonstrably non-reactive for anti-HCV, HBsAg, and anti-HBc, and negative for anti-HIV 1&2 by FDA-required testing. ApoA1 was reconstituted with Phosphate Buffered Saline 1X, pH 7.2 (PBS, Cat. No. SH30256.01, HyClone Laboratories, Logan, UT, USA).

The ApoA1 (100 μg/200 μL/mouse) was delivered intravenously via the jugular vein of mice under anesthesia with Isofluorane (2–5% at 2 L O_2_/min) as we previously described (Garbuzova-Davis et al., 2017, 2019). In Group 2, media mice received 200 μL of PBS, the identical volume as injected into ApoA1-treated mice.

### 2.4 Characteristics of disease progression

The evaluation of animal disease progression has been previously described (Garbuzova-Davis et al., 2017, 2019). Mice’s body weights were determined each week of the study. Weekly testing of grip, extension reflex, and rotarod began on week 8 and continued until 17 weeks of age.

### 2.5 Perfusion and tissue preparation

At 17 weeks of age, 4 weeks post-treatment, the ApoA1-treated, media-injected, and control mice were sacrificed for immunohistochemistry in the cervical/lumbar spinal cords for capillary permeability, glial cell expression, and ApoA1 distribution. Motor neuron survival was determined in spinal cord ventral horns by histological analyses. The mice: Group 1: *n* = 5/sex; Group 2: *n* = 5/sex, and Group 3 controls: *n* = 4/sex, received injections via the tail vein of 2% Evans blue dye (EB, Sigma-Aldrich, St. Louis, MO, USA) in a saline solution (4 mL/kg body weight) 30 min before perfusion. Spinal cord EB extravasation was evaluated in both male and female mice receiving ApoA1 vs. media and control animals as described below.

Mice were sacrificed under Euthasol® (0.22 mL/kg body weight) and perfused transcardially with 0.1 M phosphate buffer (PB, pH 7.2) followed by 4% paraformaldehyde (PFA) in PB solution under pressure-controlled fluid delivery at 80–85 mm Hg to avoid capillary rupture using previously described techniques (Garbuzova-Davis et al., 2017, 2019). Mice examined for EB leakage only received the PB solution. Post-perfusion, spinal cords were removed, post-fixed in 4% PFA for 1–2 days, and then cryopreserved overnight in 20% sucrose in 0.1 M PB. A cryostat was used to section 30 μm pieces of coronal cervical and lumbar spinal cord segments. Each fifth section was thaw-mounted on a slide, and tissue was stored at −20 °C for EB leakage, immunohistochemistry, and histology analyses.

### 2.6 Spinal cord capillary permeability

Spinal cord capillary permeability was analyzed using fluorescent EB dye (961 Da) in serial tissue sections from mice injected with EB. Analysis of dye entering the spinal cord parenchyma was performed by measuring the distance of EB extravasated from the capillary lumen. Mouse cervical and lumbar spinal cord segments from both males and females were rinsed in PBS, and then the slides were cover slipped with Vectashield® containing DAPI (Vector Laboratories, USA). An Olympus BX60 microscope was used to examine tissues under epifluorescence. The resulting images were stored for later analysis. Distance of extravasated EB was measured from the capillary lumen in the ventral spinal cords (μm) accordingly to the magnification bar using NIH ImageJ (version 1.46) software.

### 2.7 Immunohistochemical staining of astrocytes and microglia in the spinal cord

For staining astrocytes via immunohistochemistry as we previously described (Garbuzova-Davis et al., 2017), a series of cervical/lumbar spinal cord sections from randomly selected ApoA1-treated, media, and control mice (*n* = 5/ group/sex) were washed in PBS, removing the cryoprotective medium. The tissue sections were pre-incubated in a blocking solution of 10% normal goat serum (NGS) and 3% Triton 100X in PBS for 60 min at RT, followed by overnight incubation with rabbit polyclonal anti-glial fibrillary acidic protein primary antibody (GFAP, 1:500, Agilent Cat# N1506) at 4 °C. The following day, slides were washed in PBS and then incubated with a secondary antibody (goat anti-rabbit conjugated to FITC, 1:500, Alexa, Molecular Probes, USA) for 2 h at room temperature. After rinsing several times in PBS, slides were placed on coverslips with Vectashield® containing DAPI (Vector Laboratories, USA). An Olympus BX60 microscope was used to examine tissues under epifluorescence, and the resulting images were stored for later GFAP fluorescent immunodetection analysis.

In a separate set of cervical and lumbar spinal cord tissue sections from randomly selected ApoA1-treated, media, and control mice (*n* = 5/group/sex), immunohistochemical staining for microglia was performed as we described previously (Garbuzova-Davis et al., 2017). Briefly, after pre-incubating sections of tissue in a blocking solution, rabbit polyclonal anti-ionized calcium binding adapter molecule1 primary antibody (Iba-1, 1:500, FUJIFILM Wako Pure Chemical Corporation, barcode: 4987481428584, Richmond, VA, USA) was applied at 4 °C overnight. The next day, slides were rinsed in PBS and incubated with goat anti-rabbit secondary antibody conjugated to rhodamine (1:500, Alexa, Molecular Probes, USA) for 2 h at RT. After rinsing several times in PBS, slides were placed on coverslips with Vectashield® containing DAPI (Vector Laboratories, USA). An Olympus BX60 microscope was used to examine tissues under epifluorescence, and the resulting images were stored for later Iba-1 fluorescent immunodetection analysis.

### 2.8 Astrocytic and microglial immunoexpression analyses in the spinal cord

Analyzing GFAP and Iba-1 fluorescent immunodetections was performed in cervical/lumbar spinal cord ventral horns from male and female 17-week-old mice. Immunohistochemical image analyses for GFAP and Iba-1 were performed by measuring the intensity of fluorescence (%/mm^2^) in NIH ImageJ (version 1.46) software as we previously described (Garbuzova-Davis et al., 2017, 2019). For GFAP spinal cord parenchyma immunodetections in ApoA1-treated, media-injected, and control mice, immunohistochemistry images (*n* = 5 mice/group/sex, *n* = 10–12 images/cord segment) were taken from both sides of cervical/lumbar cord ventral gray matter at 40X. GFAP fluorescent intensity was determined as a percentage per mm^2^ (%/mm^2^) separately for lumbar and cervical spinal cords. Moreover, immunodetections of GFAP perivasculature were observed in the right and left sides of cervical and lumbar ventral horns from all animals. GFAP fluorescent intensities of perivascular astrocytic end-feet were determined adjoining the abluminal side of capillaries with diameters of approximately 25–30 μm. Astrocyte morphologies typical of nonreactive and reactive states were noted. Non-reactive astrocytes generally have thinner processes, while reactive astrocytes can usually be identified by their larger cell bodies and thicker, more visible processes. Similar analysis of IbA-1 fluorescent immunodetections in the cervical and lumbar spinal cords from male and female mice was performed in areas randomly selected from both sides of ventral gray matter (*n* = 5 mice/group/sex, *n* = 10–12 images/spinal cord segment). Intensities of Iba-1 fluorescence were determined separately as percentage per mm^2^ (%/mm^2^) for the cervical/lumbar spinal cords. In addition, microglial cell morphology (ramified vs. activated) was recorded.

### 2.9 Immunohistochemical staining of administered ApoA1

For validation of intravenously administered human ApoA1 distribution, immunohistochemical staining and analysis for ApoA1 were performed as we previously described (Garbuzova-Davis et al., 2022). Briefly, a series of cervical/lumbar spinal cord sections from randomly selected ApoA1-treated, media-injected, and control mice (*n* = 5/group/sex) were washed in PBS to remove the cryoprotective medium. The tissue sections were pre-incubated in a blocking solution of 10% NGS and 3% Triton 100X in PBS for 60 min at RT, followed by overnight incubation with primary anti-human recombinant rabbit monoclonal antibody (2 μg/mL, R&D Systems, Minneapolis, MN, USA, Cat. No. MAB36641) at 4 °C. The next day, slides were incubated with goat anti-rabbit secondary antibody conjugated to rhodamine (1,500, Alexa, Molecular Probes, USA) for 2 h at RT. After rinsing several times in PBS, slides were placed on coverslips with Vectashield® containing DAPI (Vector Laboratories, USA). An Olympus BX60 microscope was used to examine tissues under epifluorescence, and the resulting images were stored for later ApoA1 fluorescent immunodetection analysis.

### 2.10 Histological staining and analysis of motor neurons in the spinal cord

Cervical/lumbar spinal cord sections of randomly selected male and female mice from each group (*n* = 5/group/sex) were stained via standard protocol with 0.1% cresyl violet to examine motor neurons for Nissl substance. Motor neurons with diameters of 20–25 μm were counted in distinct levels of the lumbar (L3–L4) and cervical (C4–C6) spinal cords (*n* = 7 sections/level/spinal cord segment/group with levels about 120 μm apart) using NIH ImageJ (version 1.46) software and presented as averages per right or left spinal cord ventral horn. Analyses of motor neuron morphology were also performed in the cervical/lumbar spinal cords.

### 2.11 Statistical analysis

Data are presented as means ± S.E.M. One-way ANOVA with post-hoc Tukey HSD (Honest Significant Difference) multiple comparison test using online statistical software (astatsa.com, 2016 Navendu Vasavada) was performed for statistical analysis. Significance was defined as *p* < 0.05.

## 3 Results

ApoA1 or media were intravenously administered into male and female G93A SOD1 mice at 13 weeks of age, early symptomatic stage. At 17 weeks of age, 4 weeks after treatment, all animals were sacrificed. In total, 43 transgenic male and female G93A SOD1 mice entered the study, and three of these animals (Group 1—one male, Group 2—one male and one female) were removed due to anesthesia problems during surgeries.

### 3.1 ApoA1 effects on behavioral disease outcomes

**Figure 1:**
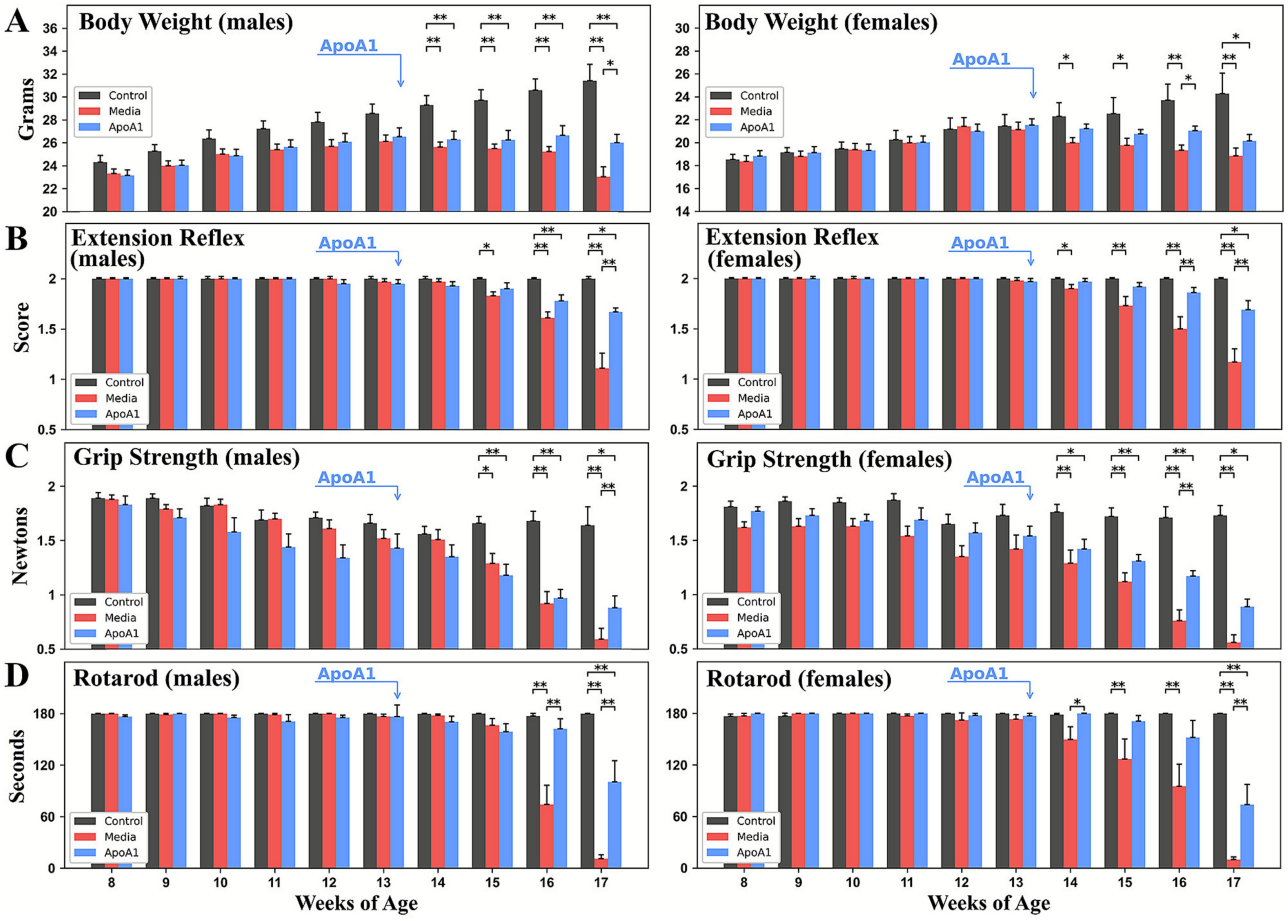
Characteristics of disease outcomes in G93A mice of both sexes receiving ApoA1 at symptomatic stage. Male and female ALS mice receiving ApoA1 at 4 weeks post-treatment significantly: **(A)** maintained body weight, **(B)** retained hindlimb extension, **(C)** delayed loss in muscle strength, and (**D**) stayed longer on rotarod vs. media-injected mice. Note that beneficial effects on motor function were determined in G93A mice of both sexes 2–3 weeks after treatment *p < 0.05, **p < 0.01.

Body weight was measured each week as an indicator of progressive muscle atrophy. Mice showed a gradual decline in body mass by approximately 13–14 weeks of age in media G93A mice of both sexes. Male mice had significantly (*p* < 0.05) higher body masses compared to media mice at 4 weeks post ApoA1 administration, whereas females maintained their significantly higher body weights at 3 weeks post-treatment (16 weeks of age, *p* < 0.05) (Figure 1A). Strikingly, at 17 weeks of age, ApoA1-treated male and female mice were about 2–3 g heavier than media-injected animals of the same sexes.

ApoA1-treated mice also performed better on functional tests. At 13 weeks of age, extension reflex scores began to worsen in media-injected G93A male and female mice (1.97 ± 0.03 and 1.98 ± 0.03 scores, respectively); scores continued to drop until 17 weeks of age (males: 1.11 ± 0.15 score; females: 1.17 ± 0.13 score). In contrast, male and female mice treated with ApoA1 showed a slower rate of decline in scores than media-injected mice. Significantly (*p* < 0.01) higher extension reflex scores of ApoA1-treated male and female vs. media mice were demonstrated at 17 weeks of age (Figure 1B). At this posttreatment time, hindlimb extension scores from ApoA1-treated males were 1.67 ± 0.04 and in females 1.69 ± 0.09.

In grip testing, G93A media animals of both sexes showed reduced muscle strength by about age 13 weeks (males: 1.52 ± 0.08 N; females: 1.42 ± 0.10 N), with progressive reductions until 17 weeks of age (males: 0.59 ± 0.10 N; females: 0.56 ± 0.07 N) (Figure 1C). These reductions in grip strength were postponed in ApoA1-treated mice, reaching significance in males (0.88 ± 0.11 N, *p* < 0.05) at 17 weeks of age and in females at 16 weeks of age (1.17 ± 0.05 N, *p* < 0.05) (Figure 1C).

Reduced performances on the rotarod were also noted in male and female media-injected mice beginning at week 13. Animals receiving ApoA1 demonstrated longer latencies than media mice. Significantly increased latencies in rotarod performance were recorded in male ALS mice after ApoA1 administration at 16 (*p* < 0.01) and 17 (*p* < 0.01) weeks of age, 3- and 4-weeks after treatment, respectively (Figure 1D). In female mice, significantly higher latency was determined at 17 weeks of age (*p* < 0.05). Importantly, 17-week-old ApoA1-treated mice showed notably higher rotarod latency (males: 100.41 ± 14.60 s; females: 73.83 ± 13.40 s) compared to media animals (males: 11.00 ± 4.58 s; females: 9.96 ± 2.26 s).

Therefore, systemic administration of ApoA1 into male and female G93A mice at the early symptomatic disease stage led to improved behavioral outcomes versus media animals by maintaining body mass and postponing declines in grip strength, hindlimb extension, and rotarod achievements.

### 3.2 ApoA1 effects on capillary permeability in the spinal cord

Capillary permeability in cervical/lumbar spinal cords was determined in tissue sections from EB-injected mice of both genders. Analysis of dye extravasated into spinal cord parenchyma was performed by measuring the distance of EB leakage from the capillary lumen in ventral spinal cords (μm) of 17-weeks-old male and female ApoA1-treated, media-injected, and control mice.

**Figure 2:**
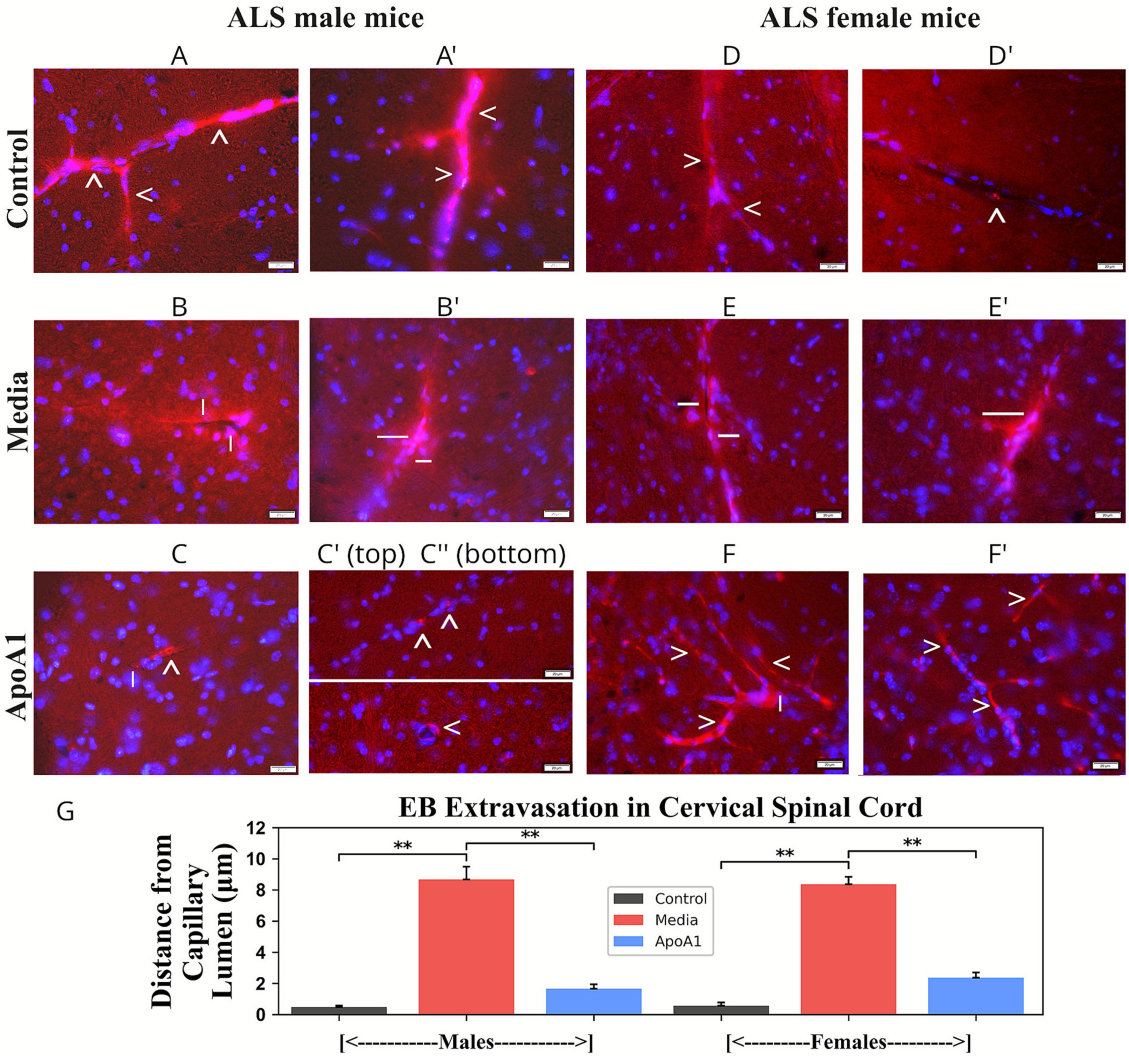
Characteristics of capillary permeability in the cervical spinal cord of G93A mice of both sexes receiving ApoA1. In cervical spinal cords, **(A,A’, arrowheads)** Evans blue dye was observed within capillaries of the ventral horn from control male mice. **(B,B’, lines)** Significantly extravasated dye was found at a distance from microvessels in the analyzed spinal cord areas of media-injected male mice. **(C,C’,C,” arrowheads)** Substantial reductions of leaky capillaries were detected in ApoA1-treated male mice. Similarly, **(D,D’, arrowheads)** control female mice displayed EB within the capillary lumen, and **(E,E’, lines)** EB leakage from microvessels was observed in media-treated female mice. ApoA1-treated female mice **(F,F’, arrowheads)** showed a significant decrease in EB extravasation into spinal cord parenchyma. Only a few microvessels were observed showing EB leakage in the spinal cord area of male **(C, line)** and female **(F, line)** mice. Scale bar in **A–F’** is 20 μm. **(G)** *Quantitative analysis of EB extravasation in the cervical ventral horn.* Analysis of EB extravasated into the spinal cord parenchyma was performed by measuring the distance of dye leakage from the capillary lumen (μm) of 17-week-old ApoA1-treated, media, and male and female control animals *p* < 0.01.

In cervical spinal cords, EB was observed within microvessels of the ventral horn from control male (Figures 2A,A’) and female (Figures 2D,D’) rodents. Evans blue leakage from microvessels was noted in ventral horns from media-injected male (Figures 2B,B’) and female (Figures 2E,E’) animals. Notably, significant (*p* < 0.01) dye extravasation was detected distant from microvessels in the analyzed mouse spinal cord sections (males: 8.67 ± 0.83 μm; females: 8.37 ± 0.47 μm) (Figure 2G). Substantially (*p* < 0.01, Figure 2G) reduced capillary leakage was noted in ventral horns of ApoA1-treated mice of both genders: males: 1.66 ± 0.28 μm (Figures 2C,C’,C”) and females: 2.36 ± 0.35 μm (Figures 2F,F’).

**Figure 3:**
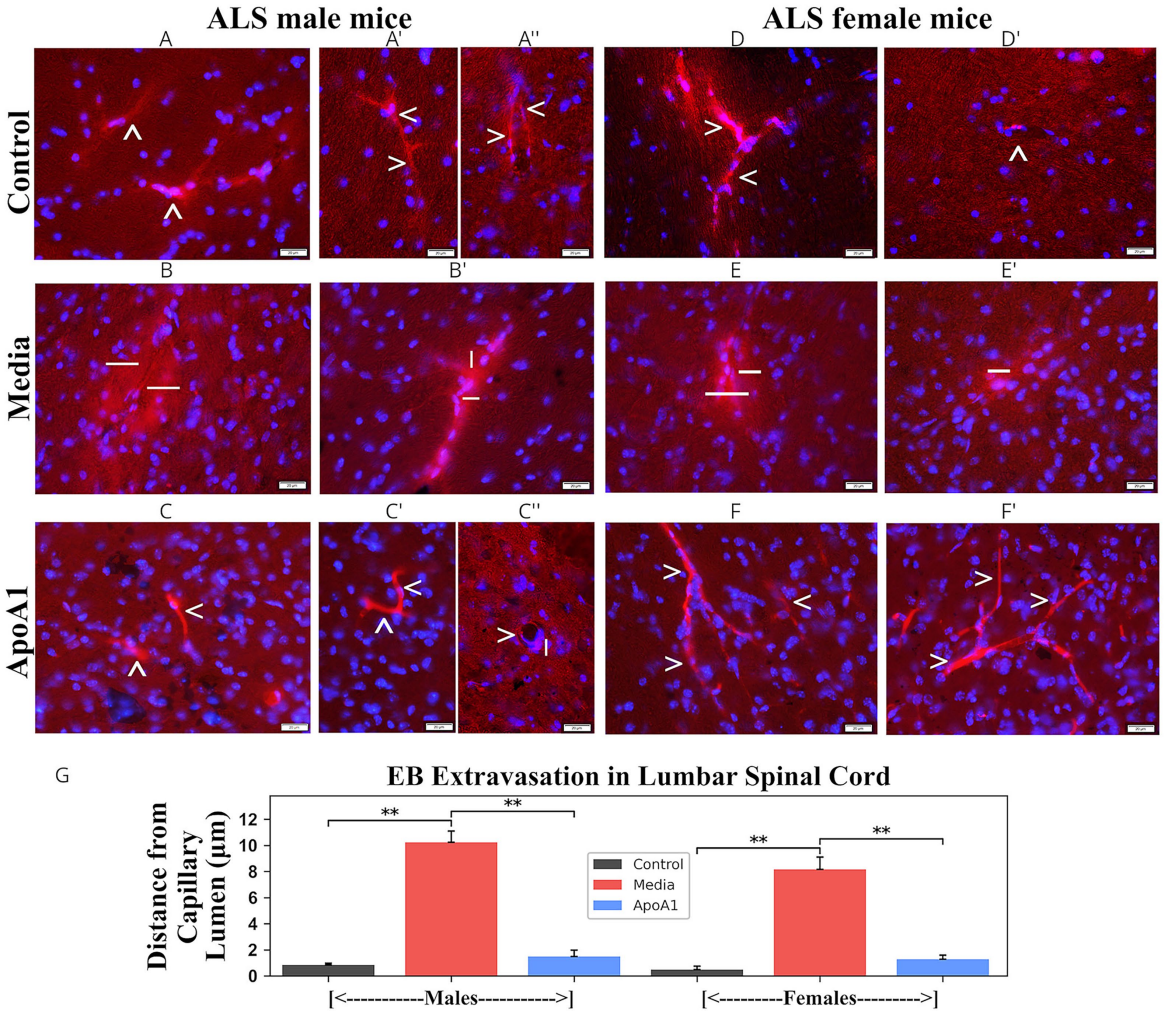
Characteristics of capillary permeability in the lumbar spinal cord of G93A mice of both sexes receiving ApoA1. In lumbar spinal cords, **(A,A’,A”, arrowheads)** Evans blue was clearly detected within the capillaries of the ventral horn in control male mice. **(B,B’, lines)** EB diffused extensively into the ventral horn parenchyma from many blood vessels in media male mice. **(C,C’,C”, arrowheads)** Significantly reduced capillary permeability for EB was detected in ApoA1-treated male mice. **(D,D’, arrowheads)** Analogous to finding in control male mice, control female mice showed EB within the capillary lumen. **(E,E’, lines)** EB leakage from microvessels was observed in media-treated female mice. **(F,F’, arrowheads)** No EB leakage from microvessels into spinal cord parenchyma was observed in ApoA1-treated female mice. Scale bar in **A–F’** is 20 μm. **(G)** *Quantitative analysis of EB extravasation in the lumbar ventral horn.* Analysis of EB extravasated into the spinal cord parenchyma was performed by measuring the distance of dye leakage from the capillary lumen (μm) of 17-weeks-old ApoA1-treated, media, and control male and female animals *p* < 0.01.

Microvessels leaking EB were sparse in this spinal cord area (Figures 2C,F). In lumbar spinal cord segments, EB was detected in ventral horn capillaries of both control male (Figures 3A,A’,A”) and female (Figures 3D,D’) mice, similarly to cervical findings. EB diffusion (*p* < 0.01, Figure 3G) into the ventral horn of spinal cord parenchyma was identified from multiple microvessels in media male (Figures 3B,B’) and female (Figures 3E,E’) mice at large distances (males: 10.24 ± 0.85 μm; females: 8.16 ± 0.94 μm) (Figure 3G). Significant (*p* < 0.01, Figure 3G) reductions in ventral horn capillary permeability were noted in ApoA1-treated male (1.49 ± 0.48 μm, Figures 3C,C’,C”) and female (1.28 ± 0.31 μm, Figures 3F,F’) animals. Similar to cervical results, EB extravasation was identified in some ApoA1-treated ALS mice microvessels (Figure 3C”).

Consequently, improved capillary impermeability by significantly reduced EB leakage in G93A SOD1 ApoA1 male and female mouse spinal cords links to potential restoration of the blood–spinal cord barrier in treated mice, leading to enhancement of beneficial behavioral disease outcomes.

### 3.3 ApoA1 effects on glial cells in the spinal cord

Immunohistochemical analyses of astrocytes and microglia in ventral horns of the cervical/lumbar spinal cords of 17-weeks-old ApoA1-treated, media, and control mice at 17 weeks of age of both genders were accomplished by fluorescence immunostaining with GFAP and Iba-1, respectively.

**Figure 4:**
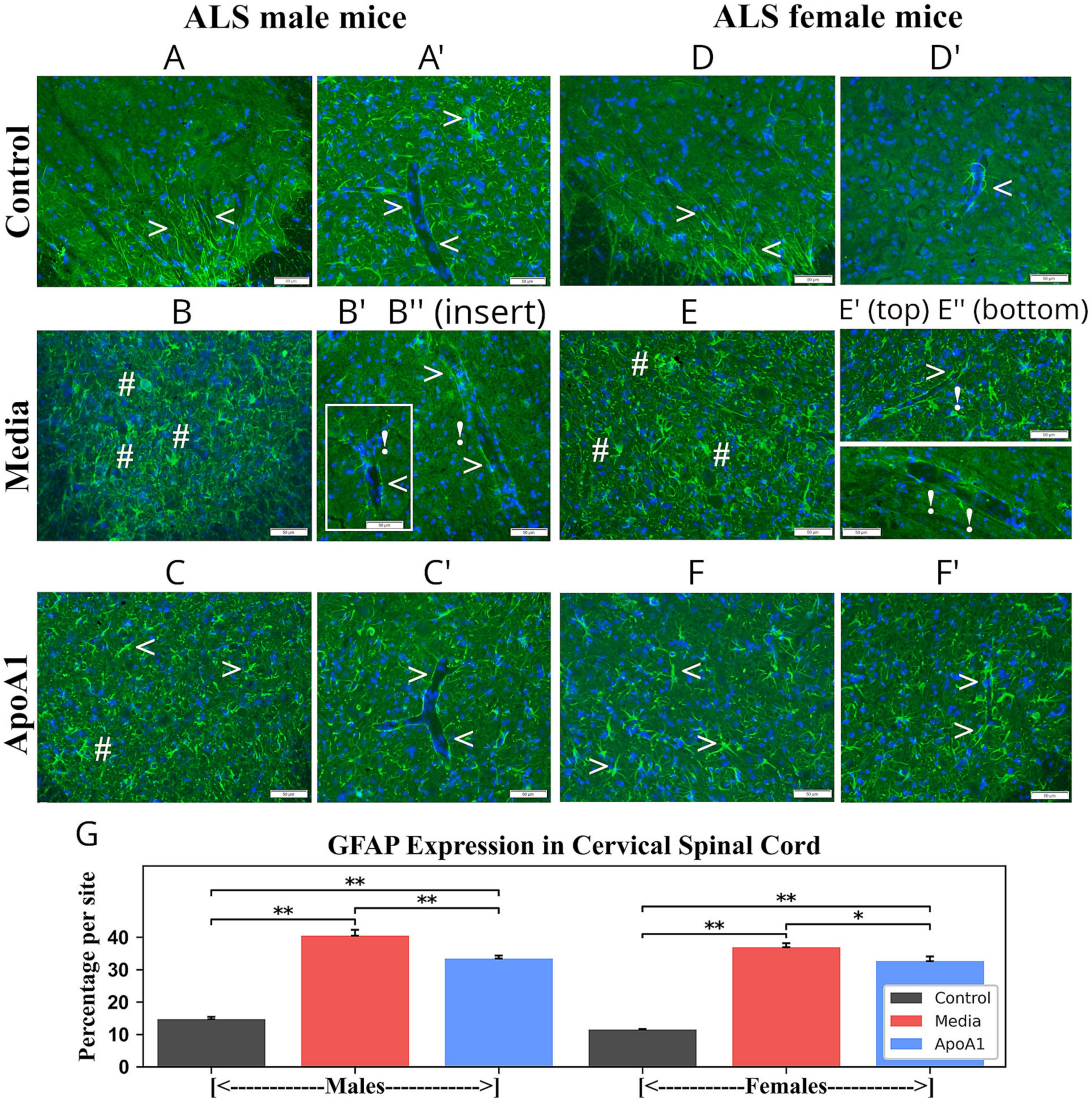
Characteristics of astrocytes in the cervical spinal cord of G93A mice of both sexes receiving ApoA1. In the cervical spinal cord, **(A, arrowheads)** astrocytes with fine cellular processes in spinal cord parenchyma and **(A’, arrowheads)** perivascular astrocytes fully covering capillaries were detected in male control mice. **(B, hashmarks)** Astrocytes with large cell bodies and hypertrophic processes in addition **(B’,B”, arrowheads, exclamation mark)** to capillaries partly surrounded by perivascular astrocytes were observed in media male mice. ALS-treated males **(C, arrowheads)** showed astrocytes with normal morphology, and **(C’, arrowheads)** typical perivascular astrocytes with capillary coverage. **(D, arrowheads)** Standard appearance of parenchymal astrocytes and **(D’, arrowheads)** perivascular astrocytes in the ventral horns was determined in control female mice. Media-treated female mice **(E, hashmarks)** displayed many hypertrophic astrocytes with **(E’,E”, exclamation mark)** perivascular astrocytes moderately covering capillaries. **(F, arrowheads)** Astrocytes with typical morphology and **(F’, arrowheads)** capillary delineated perivascular astrocytes were detected in ApoA1-treated female mice. Scale bar in **A–F’** is 50 μm. **(G)** *Quantitative analysis of GFAP immunoexpression in the cervical ventral horn*. Immunohistochemical analyses of astrocytes in the ventral horns of the cervical spinal cords from control, media-injected, and ApoA1-treated mice at 17 weeks of age of both sexes were performed by measuring the intensity of fluorescent GFAP expression (%/mm^2^/site) *p < 0.05, **p < 0.01.

Cervical spinal cord astrocytes with fine cellular processes were detected in male (Figure 4A) and female (Figure 4D) control mice, while media animals of both genders (Figures 4B,E) presented large astrocytes with hypertrophic processes. Moreover, capillaries in media male (Figures 4B,B’) and female (Figures 4E,E”) mice were partly surrounded by astrocytes. In contrast, healthy-appearing astrocytes in the perivasculature completely covered control male (Figure 4A’) and female (Figure 4D’) mice capillaries. In ALS mice of both genders (Figures 4C,F) treated with ApoA1, astrocytes displayed slender cell processes resembling normal morphology in ventral horns. Only a few morphologically reactive astrocytes were detected. Additionally, many microvessels displayed typical perivascular astrocyte coverage in both male (Figure 4C’) and female (Figure 4F’) spinal cords from ApoA1-treated animals. Quantified GFAP immunodetection corresponded with astrocyte morphology in the cervical ventral horns of evaluated mice, revealing a significant (*p* < 0.01) increase in GFAP fluorescence in media-injected rodents (males: 40.44 ± 1.84%; females: 36.90 ± 1.26%) vs. controls (males: 14.68 ± 0.78%; females: 11.49 ± 0.16%) (Figure 4G). Significantly reduced GFAP immunodetection was noted in male (33.38 ± 0.94%, *p* < 0.01) and female (32.60 ± 1.44%, *p* < 0.05) mice treated with ApoA1 compared to media animals.

**Figure 5:**
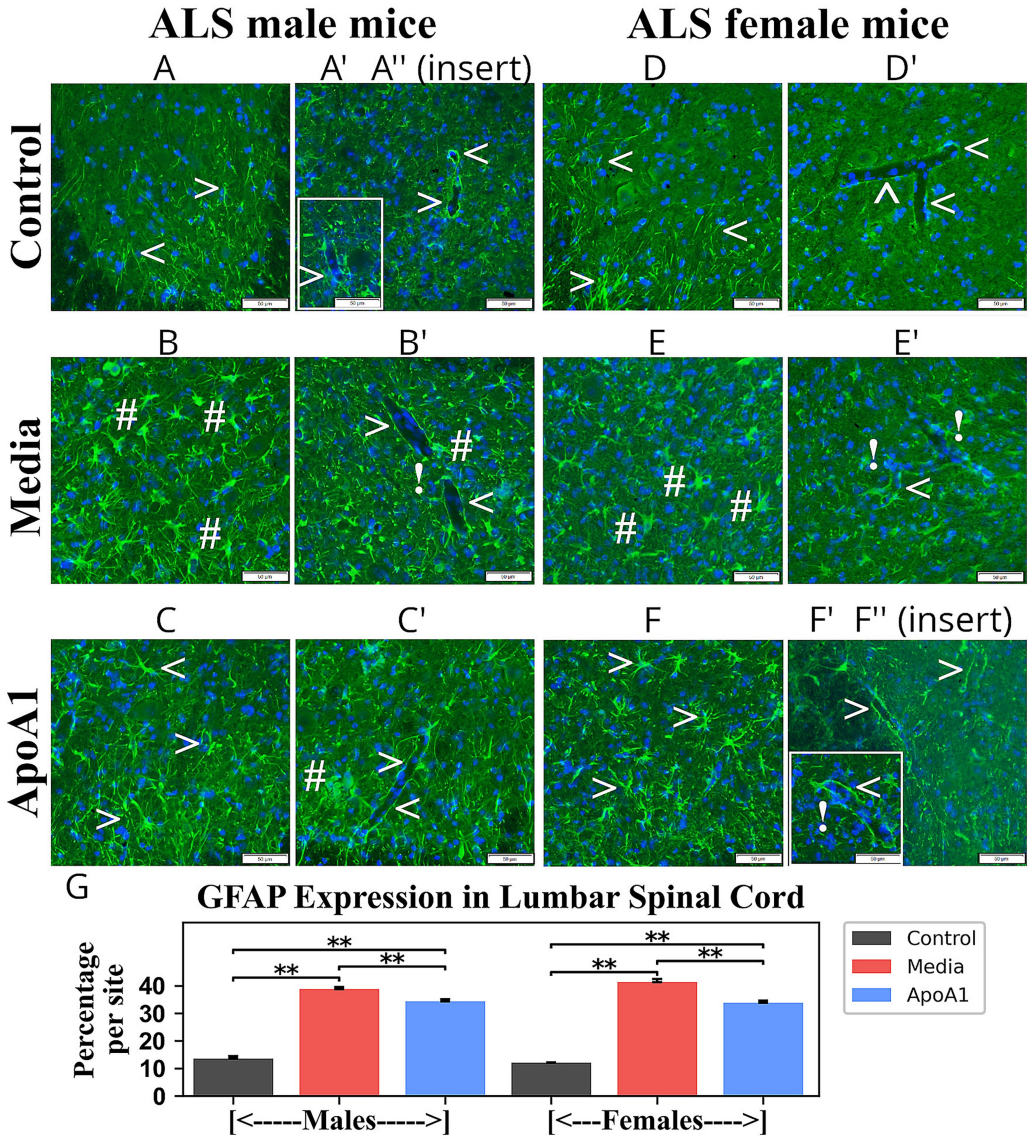
Characteristics of astrocytes in the lumbar spinal cord of G93A mice of both sexes receiving ApoA1. In the lumbar spinal cord, **(A, arrowheads)** astrocytes with normal morphology and **(A’,A”, arrowheads)** capillaries fully covered by perivascular astrocytes were detected in the spinal cord parenchyma of male control mice. Media-treated ALS male mice **(B, hashmarks)** demonstrated astrocytes with large cell bodies and thick processes, and **(B’, arrowheads, exclamation mark)** diminished perivascular astrocyte coverage of capillaries. **(C, arrowheads)** Astrocytes with typical morphology and **(C’, arrowheads)** capillaries fully covered by perivascular astrocytes were observed in ApoA1-treated males. **(D, arrowheads)** Distinctively typical parenchymal astrocytes and **(D’, arrowheads)** perivascular astrocytes in the ventral horn were determined in control female mice. Media-treated female mice **(E, hashmarks)** displayed many hypertrophic astrocytes with **(E’, exclamation mark)** perivascular astrocytes partially covering capillaries. **(F, arrowheads)** Astrocytes with fine cell processes and **(F’, arrowheads)** perivascular astrocytes with clear surrounding capillaries were detected in ApoA1-treated female mice. **(F”, exclamation mark)** Only a few capillaries were observed with diminished perivascular astrocytes. Scale bar in **A–F”** is 50 μm. **(G)** *Quantitative analysis of GFAP immunoexpression in the lumbar ventral horn*. Immunohistochemical analyses of astrocytes in the ventral horns of the lumbar spinal cords from control, media-injected, and ApoA1-treated mice at 17 weeks of age of both sexes were performed by measuring the intensity of fluorescent GFAP expression (%/mm^2^/site) **p < 0.01.

As in cervical spinal cords, astrocyte morphologies were determined in lumbar spinal cords from 17-week-old Apoa1-treated, media, and control mice of both genders. ALS media-injected male (Figure 5B) and female (Figure 5E) mice displayed astrocytosis, with larger cell bodies and thicker processes than controls of both genders (Figures 5A,D). Furthermore, diminished perivascular astrocyte coverage of capillaries was detected in these mice (Figures 5B,E) vs. controls (Figures 5A’,A”,D’). In ApoA1-treated male (Figure 5B) and female (Figure 5E) mice, morphologically reactive astrocytes were substantially sparser in the ventral horns. Thin cell processes were found in numerous astrocytes. Perivascular astrocytes also appeared with normal morphology (Figures 5C’,F’,F”) similar to controls. Measured GFAP immune-expression in ventral horns of lumbar spinal cords showed significantly (*p* < 0.01) increased fluorescence in media-injected animals (males: 38.77 ± 0.81%; females: 41.33 ± 1.10%) vs. controls (males: 13.56 ± 0.83%; females: 12.08 ± 0.14%) (Figure 5G). Whereas, a significant (*p* < 0.01) reduction in GFAP immunodetection was detected in ApoA1-treated male (34.39 ± 0.75%) and female (33.81 ± 0.80%) animals (Figure 5G).

**Figure 6:**
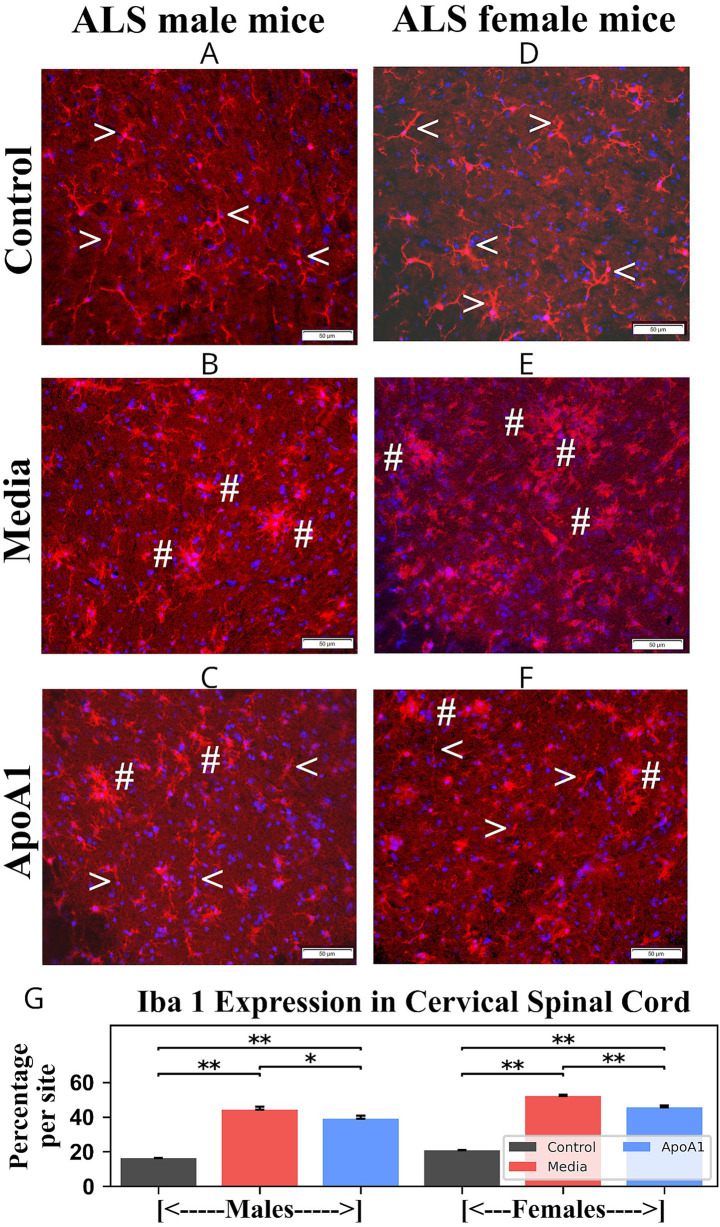
Characteristics of microglia in the cervical spinal cord of G93A mice of both sexes receiving ApoA1. In the cervical spinal cord, **(A, arrowheads)** microglia with fine cellular processes were detected in the ventral horns of control male mice. **(B, hashmarks)** Microglial cells with large cell bodies and thick processes were determined in media-injected male mice. **(C)** Microglial cells with normal morphology (**arrowheads**) were observed in ApoA1-treated male mice; fewer microglial cells with large cell bodies and short processes (**hashmarks**) were seen in these treated mice. **(D, arrowheads)** Microglial cells with typical morphology were present in control female mice. **(E, hashmarks)** Numerous microglial cells with large cell bodies were observed in media females. **(F)** Microglial cells with fine cellular processes (**arrowheads**), in addition to some cells with large cell bodies (**hashmarks**), were determined in treated ALS females. Scale bar in A–F is 50 μm. **(G)** *Quantitative analysis of Iba-1 immunoexpression in the cervical ventral horn.* Immunohistochemical analyses of microglia in the ventral horns of the cervical spinal cords from control, media-injected, and ApoA1-treated mice at 17 weeks of age of both sexes were performed by measuring the intensity of fluorescent Iba-1 expression (%/mm^2^/site) *p < 0.05, **p < 0.01.

Additionally, immunohistochemical analyses of microglial cell statuses in the cervical/lumbar spinal cords from 17-weeks-old ApoA1-treated, media, and control rodents of both genders were accomplished through fluorescence immunostaining with anti-Iba-1 antibody. In cervical spinal cords, control male (Figure 6A, 1.63 ± 0.11%) and female (Figure 6D, 2.09 ± 0.14%) mice presented microglial cells with thin processes. In contrast, microglia with larger bodies and thicker processes were identified at a significantly (*p* < 0.01) higher density in ventral horns from media male (Figure 6B, 44.33 ± 1.72%) and female (Figure 6E, 52.08 ± 0.77%) mice compared to controls. In ALS ApoA1-treated animals, moderately decreased Iba-1 immunodetection was found in the ventral horn of males (Figure 6C, 38.92 ± 2.01%, *p* < 0.05) and females (Figure 6F, 45.57 ± 1.07%, *p* < 0.01). Fewer microglia, typical of the activated state, cells with large bodies and short processes, were noted in the cervical spinal cords of these treated ALS mice. Moreover, increased microglial cells were seen with normal morphology.

**Figure 7:**
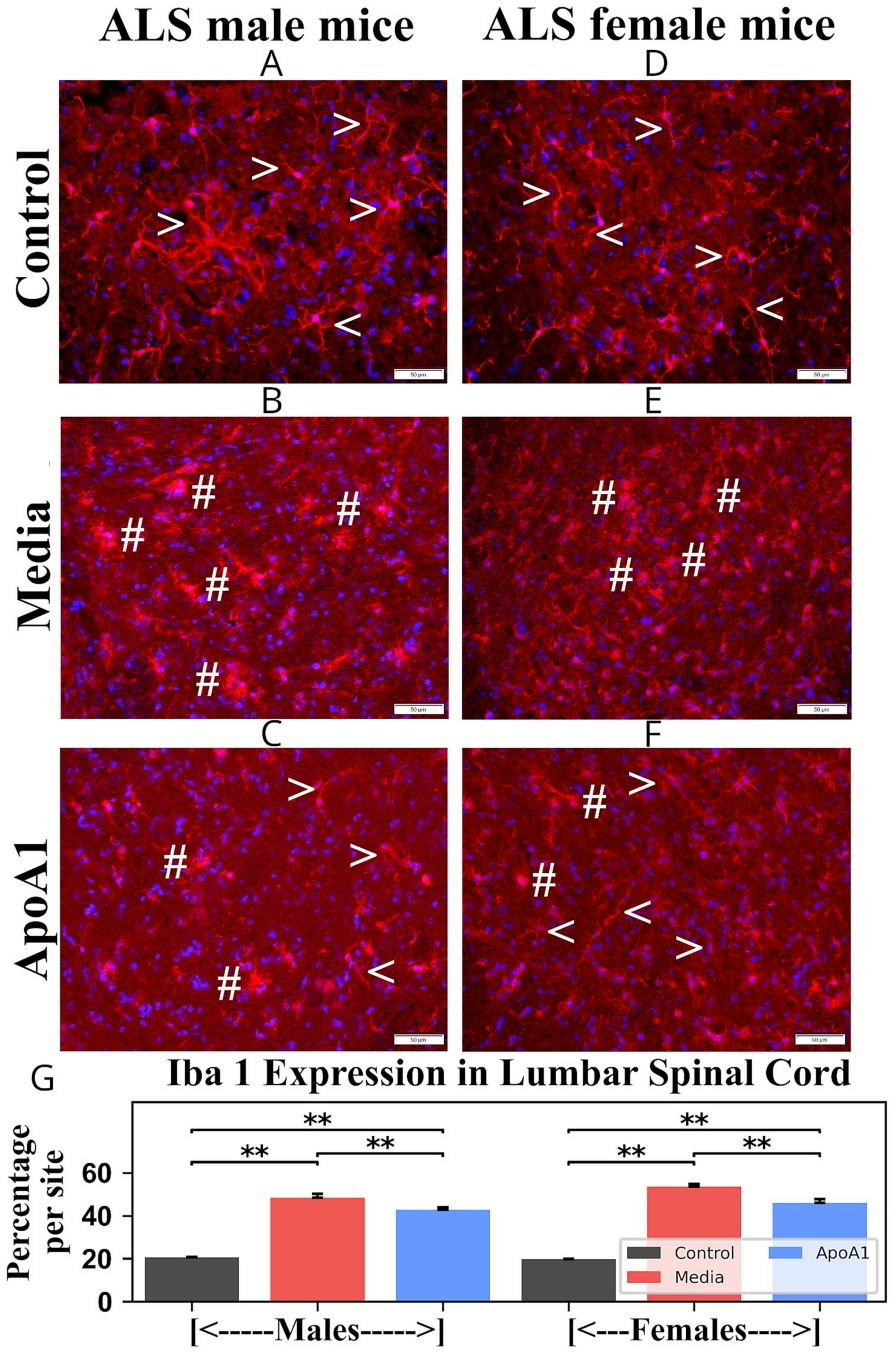
Characteristics of microglia in the lumbar spinal cord of G93A mice of both sexes receiving ApoA1. In the lumbar spinal cord, **(A, arrowheads)** microglia with typical cell morphology were identified in the ventral horns of control male mice. **(B, hashmarks)** Numerous microglial cells with large cell bodies were determined in male mice. **(C)** Many ramified cells with smaller cell bodies and fine cell processes (**arrowheads**), in addition to some activated microglial cells (**hashmarks**), were observed in male mice treated with ApoA1. **(D, arrowheads)** Microglial cells with fine cellular processes were detected in control female mice. **(E, hashmarks)** Microglial cells with large cell bodies and thick processes were observed in media-treated females. **(F)** Microglial cells with small cell bodies and fine cellular processes (**arrowheads**), as well as a few cells with large cell bodies (**hashmarks**), were determined in ApoA1-treated females. Scale bar in A–F is 50 μm. **(G)** *Quantitative analysis of Iba-1 immunoexpression in the lumbar ventral horn*. Immunohistochemical analyses of microglial cells in the ventral horns of the lumbar spinal cords from control, media-injected, and ApoA1-treated mice at 17 weeks of age of both sexes were performed by measuring the intensity of fluorescent Iba-1 expression (%/mm^2^/site) **p < 0.01.

Analogously to cervical findings, microglia were analyzed in lumbar spinal cords of 17-week-old ApoA1-treated, media, and control animals. Microgliosis was identified in ventral horns of ALS media-treated rodents, through many microglia with larger bodies and thicker processes in male (Figure 7B, 48.42 ± 1.81%, *p* < 0.01) and female (Figure 7E, 53.52 ± 1.21%, *p* < 0.01) mice, compared to controls (males: 2.07 ± 0.16%; females: 1.97 ± 0.12%) (Figures 7A,D) with typical cell morphology. In ApoA1-treated male (Figure 7C) and female (Figure 7F) mice, microglial cells with morphologies typical of activation were significantly (p < 0.01) reduced in the ventral horns to 42.80 ± 1.23% (males) and 45.97 ± 1.74% (females) (Figure 7G), respectively. Numerous ramified microglia were found in these mice with small cell bodies and thin processes.

Thus, analyses of GFAP and Iba-1 immunodetections in cervical/lumbar spinal cords displayed significant astro- and microgliosis in 17-week-old media animals of both genders. Furthermore, diminished perivascular astrocytes surrounded by capillaries were detected in these mice. After ApoA1 administration, morphological signs of astrocyte cell reactivity and microglial cell activation were significantly reduced in both male and female mice. Importantly, re-established perivascular astrocytes were identified in many spinal cord microvessels in ApoA1-treated animals of both genders.

### 3.4 Validation of administered ApoA1 in the spinal cord

**Figure 8:**
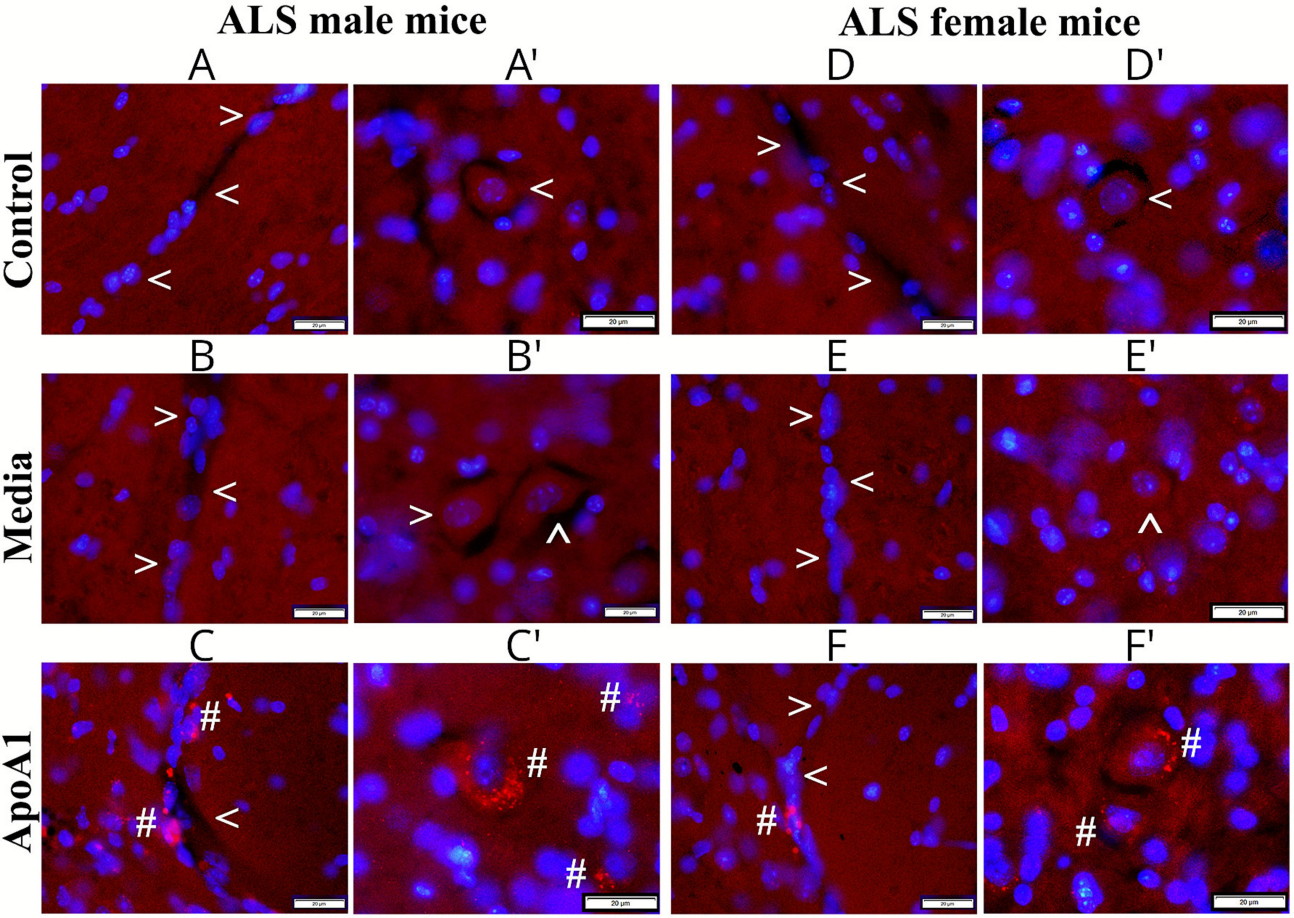
Immunohistochemical validation of ApoA1 in the cervical spinal cord of G93A mice of both sexes. In the cervical spinal cord, ApoA1 was not detected within the capillary lumen or motor neurons in control **(A,A’, arrowheads)** or media **(B,B’, arrowheads)** male mice. ApoA1-treated male animals demonstrated positive protein immunostaining within numerous microvessels **(C, hashmarks)** and motor neurons **(C’, hashmarks)**. Also, ApoA1 was determined in the capillary endothelium or outside of microvessels. Similarly, negative immunostaining for ApoA1 was observed in the capillary lumen and motor neurons in control **(D,D’, arrowheads)** and media **(E,E’, arrowheads)** female mice. Definite ApoA1 immunoreactivity was detected within capillaries **(F, hashmarks)** and motor neurons **(F’, hashmarks)** of treated females. Scale bar in **A–F’** is 20 μm.

Immunohistochemistry analysis of systemically delivered human ApoA1 was accomplished in cervical/lumbar spinal cords of 17-weeks-old ApoA1, media, and control rodents of both genders. In the cervical spinal cord. ApoA1 was not detected within the capillary lumen or motor neurons in control and media male (Figures 8A,A’,B,B’) and female (Figures 8D,D’,E,E’) mice. Controversially, ApoA1-treated male (Figures 8C,C’) and female (Figures 8F,F’) animals demonstrated positive protein staining within numerous microvessels and motor neurons. Interestingly, ApoA1 was noticed in capillary endothelium or outside of microvessels.

**Figure 9:**
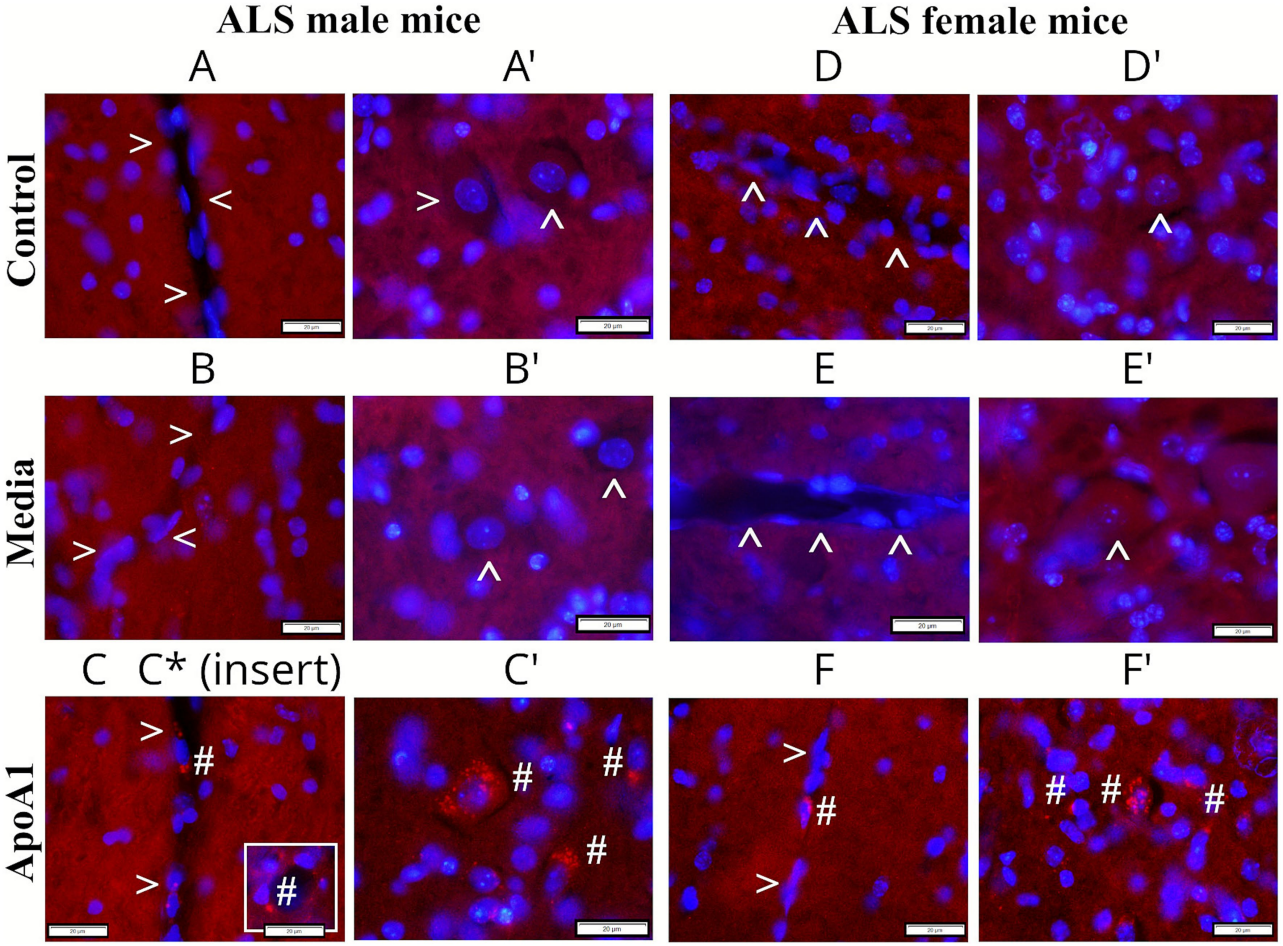
Immunohistochemical validation of ApoA1 in the lumbar spinal cord of G93A mice of both sexes. In the lumbar spinal cord, ApoA1 was not detected within the capillary lumen or motor neurons in control **(A,A’, arrowheads)** and media **(B,B’, arrowheads)** male mice. Positive ApoA1 immunostaining was observed within microvessels (C,C*, hashmarks) and motor neurons **(C’, hashmarks)** in treated male mice. ApoA1 was negatively immunostained within microvessels and motor neurons in control **(D,D’, arrowheads)** and media **(E,E’, arrowheads)** female mice. Positive ApoA1 immunostaining was detected within capillaries **(F, hashmarks)** and motor neurons **(F’, hashmarks)** of treated females. Scale bar in **A–F’** is 20 μm.

Similarly to the cervical spinal cord, negative ApoA1 staining was observed in the lumbar spinal cord capillaries and motor neurons from control and media male (Figures 9A,A’,B,B’) and female (Figures 9D,D’,E,E’) animals. In ApoA1-treated male (Figures 9C,C’) and female (Figures 9F,F’) mice, ApoA1 was detected in the capillary lumen and motor neurons. Furthermore, the protein was seen outside of capillaries (Figure 9C*) or disseminated in lumbar cord parenchyma near motor neurons (Figure 9F’). Together, validation of administered human ApoA1 into ALS mice of both genders showed protein distribution within microvessels and motor neurons in the cervical and lumbar spinal cords of treated animals, whereas negative ApoA1 staining was observed in control and media-treated male and female mice.

### 3.5 ApoA1 effects on motor neuron survival in the spinal cord

Sets of cervical/lumbar spinal cord sections from ApoA1, media-injected, and control animals were stained with cresyl violet (Nissl body) to examine status of motor neurons. Motor neuron counts were performed in mice of both genders at 17 weeks of age in distinct ventral horn levels of C4–C6 cervical and L3–L4 lumbar spinal cords.

**Figure 10:**
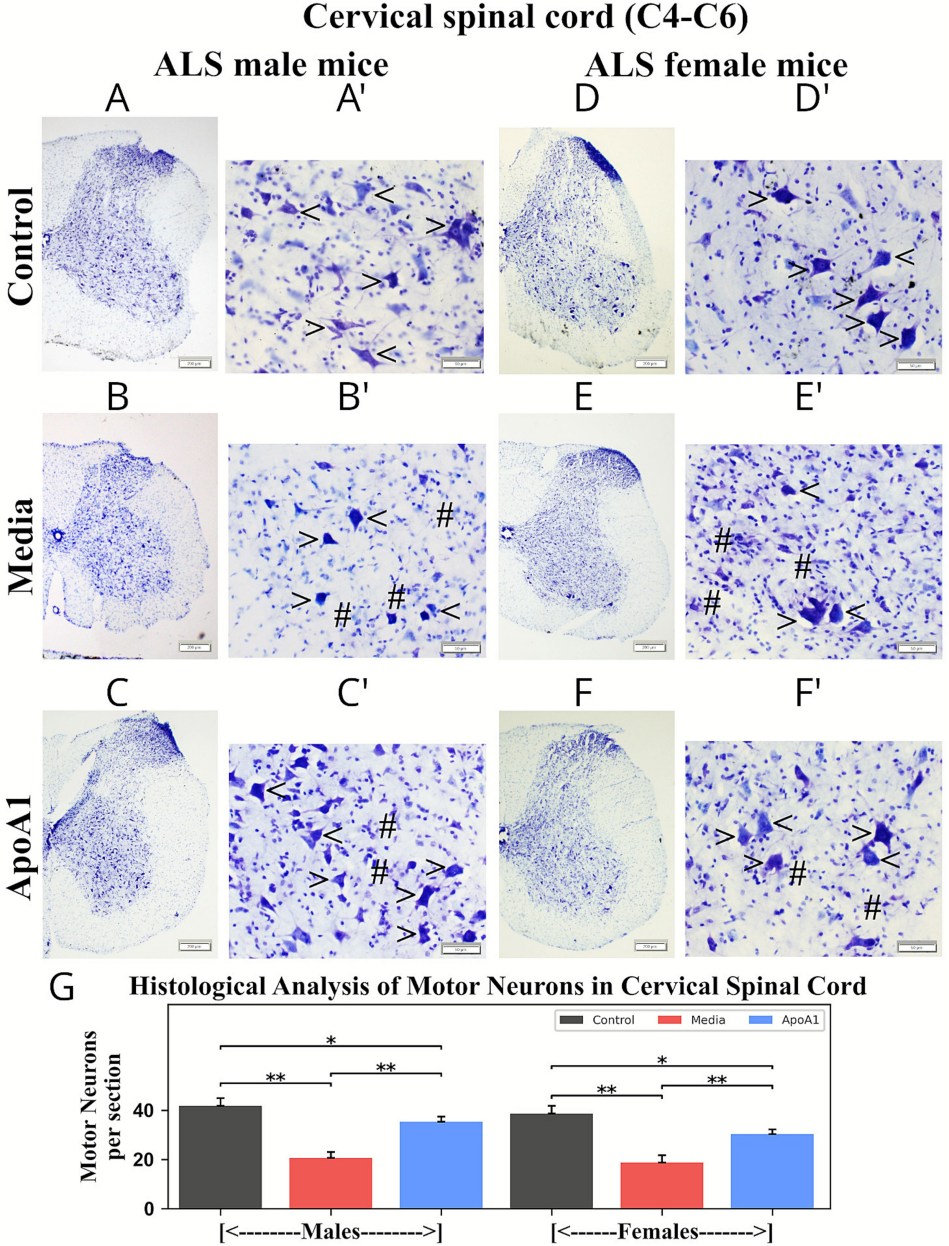
Characteristics of motor neurons in the cervical spinal cord (C4–C6) of G93A mice of both sexes receiving ApoA1. In the cervical spinal cord, **(A,A’, arrowheads)** healthy motor neurons with large soma and neuritic processes were determined in the ventral horns of control male mice. **(B,B’)** Most motor neurons had degenerated (**hashmarks**), and only a few motor neurons appeared healthy (**arrowheads**) in media-treated male mice. **(C,C’)** ApoA1-treated male mice demonstrated robust motor neurons with large soma (**arrowheads**), with some degenerated cells (**hashmarks**). **(D,D’, arrowheads)** Numerous healthy motor neurons were visible in control female mice. **(E,E’)** Media-treated female mice showed mostly degenerated motor neurons (**hashmarks**), and some cells were morphologically vigorous (**arrowheads**). **(F,F’)** More healthy motor neurons (**arrowheads**) appeared in the ventral horns of treated female mice; a few cells were visibly degenerated (**hashmarks**). Scale bar in **A–F** is 200 μm, **A’–F’** is 50 μm. **(G)** *Histological analysis of motor neurons in cervical spinal cords.* Motor neuron counts were performed at discrete levels of the cervical spinal cords (C4–C6) from control, media-injected, and ApoA1-treated mice at 17 weeks of age, of both sexes, and are presented as averages per ventral horn for both spinal cord sides *p < 0.05, **p < 0.01.

In controls, large soma and neuritic processes indicated robust health of motor neurons in cervical spinal cords of male (Figures 10A,A’) and female (Figures 10A,D’) animals. In media male (Figures 10B,B’) and female (Figures 10E,E’) mice, most motor neurons had degenerated or vacuolated. Normal appearing motor neurons were sparse in media mice ventral horns. Significantly (*p* < 0.01) reduced survival of motor neurons was determined in media-injected animals (males: 20.72 ± 2.51%; females: 18.83 ± 2.90%) vs. controls (males: 41.81 ± 3.33%; females: 38.77 ± 3.11%) (Figure 10G). ALS male (Figures 10C,C’) and female (Figures 10F,F’) mice receiving ApoA1 demonstrated robust appearing motor neurons with significantly (*p* < 0.01) higher motor neuron numbers (males: 35.33 ± 3.12%; females: 30.34 ± 1.92%) compared to media mice (Figure 10G). Although numerous healthy, large soma, cervical–spinal–cord motor neurons were identified, some degenerated motor neurons were visible.

**Figure 11:**
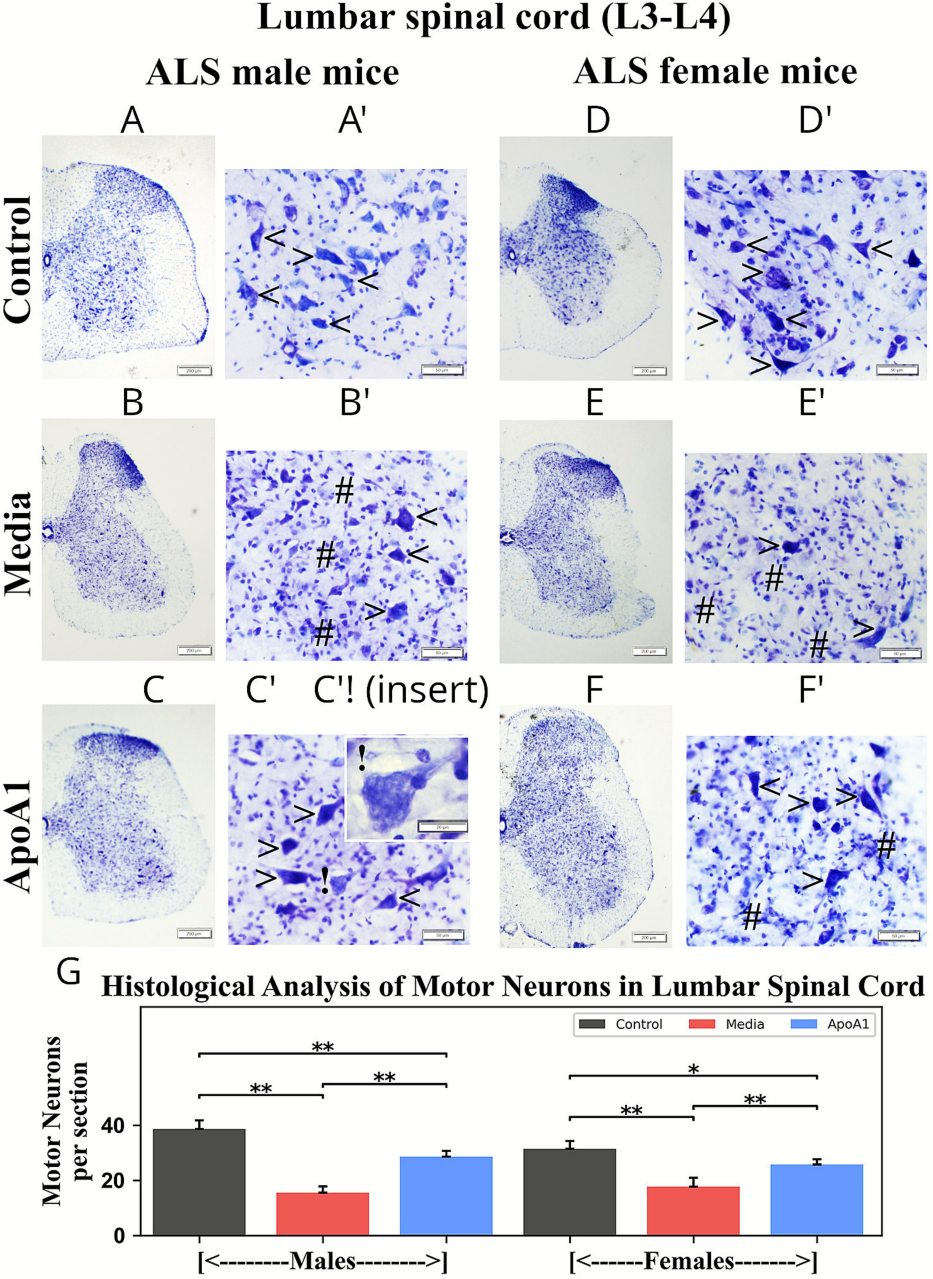
Characteristics of motor neurons in the lumbar spinal cord (L3–L4) of G93A mice of both sexes receiving ApoA1. In the lumbar spinal cord, **(A,A’, arrowheads)** healthy motor neurons with large soma were noted in the ventral horns of control male mice. **(B,B’)** Many degenerated motor neurons (**hashmarks**) with only a few surviving (**arrowheads**) were seen in media-treated male mice. **(C,C’, arrowheads)** Numerous healthy motor neurons were determined in ApoA1-treated male mice. **(C’!)** Some healthy motor neurons with numerous particles were identified. **(D,D’, arrowheads)** Mostly healthy motor neurons were visible in control female mice. **(E,E’)** Degenerated motor neurons (**hashmarks**) and some healthy cells (**arrowheads**) were detected in media-treated female mice. **(F,F’)** Healthy motor neurons (**arrowheads**) and some degenerated cells (**hashmarks**) were detected in the ventral horns of ApoA1-treated female mice. Scale bar in **A–F** is 200 μm, **A’–F’** is 50 μm, **C’!** is 20 μm. **(G)** *Histological analysis of motor neurons in lumbar spinal cords.* Motor neuron counts were performed at discrete levels of the lumbar spinal cords (L3–L4) from control, media-injected, and ApoA1-treated mice at 17 weeks of age, of both sexes, and are presented as averages per ventral horn for both spinal cord sides *p < 0.05, **p < 0.01.

Similarly, a significant (*p* < 0.01) reduction in motor neuron numbers was noted in lumbar spinal cords from media male (15.55 ± 2.32%) and female (17.76 ± 3.23%) mice vs. controls (males: 38.72 ± 3.311%; females: 31.51 ± 2.82%) (Figure 11G). These media-treated mice of both genders (Figures 11B,B’,E,E’) showed many degenerated motor neurons with only a few surviving. Male (Figures 11C,C’) and female (Figures 11F,F’) mice treated with ApoA1 presented many healthy motor neurons and significantly (*p* < 0.01) greater cell survival compared to media animals. Interestingly, some healthy motor neurons containing numerous unidentified particles were found in male mice (Figure 11C’!). Motor neuron numbers in ApoA1-treated male and female mice were 28.64 ± 2.13% and 25.88 ± 1.91%, respectively (Figure 11G).

Thus, histological analysis of motor neurons in cervical/lumbar spinal cord ventral horns showed notably fewer motor neurons in 17-weeks-old media male and female animals. Improved survival of motor neurons was found in cervical/lumbar spinal cords from male and female mice treated with ApoA1 at 4 weeks post-injection.

## 4 Discussion

Our current investigation evaluated the results of ApoA1 systemically administered into male and female G93A SOD1 mice at the early disease stage towards enhancing BSCB integrity. Study findings revealed that ApoA1 effectively: (1) postponed behavioral indicators of disease progress; (2) reduced EB leakage into the parenchyma of the spinal cord; (3) decreased gliosis; (4) incorporated into endothelium and motor neurons; and (5) increased survival of spinal cord motor neurons in both males and females. These novel data are evidence that ApoA1 administration benefits ALS mice of both genders, probably by restorative effects on disease-damaged microvessels with possibilities of BSCB repair. The results of our preclinical study suggest that ApoA1 may be a potential protein therapeutic for restoring vascular integrity in ALS.

Dyslipidemia in ALS, as an important factor in the pathologic disease process, results from dysregulation of lipoprotein metabolism (Mariosa et al., 2017; Chaves-Filho et al., 2019; González De Aguilar, 2019; Chełstowska et al., 2021; Xia et al., 2023; Chalitsios et al., 2024). Since a reduced LDL/HDL ratio has been observed in ALS patients, treatment with ApoA1 might increase HDL-mediated removal of cholesterol from cells and improve the cholesterol efflux process [reviewed in Cochran et al. (2021), and Bhale and Venkataraman (2022)]. Although confirmation of cholesterol levels in ALS mice before and after ApoA1 treatment is needed and will be addressed in our future study, beneficial protein treatment effects augment delayed disease progression and motor neuron survival in G93A SOD1 mice of both genders. A recently published review (Rajha et al., 2025) comprehensively discussed the role of various constituents of apolipoprotein A in a number of neurological disorders, including ALS. The authors emphasized the functional role of ApoA1 as an essential HDL component, exerting anti-inflammatory and neuroprotective effects. In terms of neuroprotection, ApoA1 reduced EC death via P13K/Akt signaling, supporting BBB integrity, and limiting leukocyte infiltration into the CNS. Although the present study did not investigate mechanisms of ApoA1 effects, contributory, including anti-oxidative, pathways will be investigated in a future study. Additionally, since ApoA1 may modulate cytokine production in immune cells, this possibility will also be explored. Of note, while ApoA1 often binds to ABCA1 or LCAT proteins for lipid transport and HDL-related functions, it also has direct biologically active capabilities independent of such interactions. However, ApoA1’s actions in regard to motor neurons or the neurovasculature in ALS, whether dependent or independent of protein binding, could have significant therapeutic benefits.

Our primary focus in the current study was to evaluate ApoA1’s therapeutic potential on enhancing capillary EC utility towards restoring altered BSCB in ALS. Previously, we showed (Garbuzova-Davis et al., 2022) that ApoA1 at a dose of 100 μg substantially reduced EC death subjected to pathologic conditions reminiscent of ALS *in vitro*. Using the same dose of ApoA1 in the treatment of symptomatic ALS mice, results demonstrated substantially reduced EB leakage into the parenchyma of cervical and lumbar spinal cords in ALS mice of both genders. Mainly, injected EB dye was detected within ventral horn capillaries of ApoA1-treated male and female mice, suggesting probable restoration of BSCB that leads to enhancement of beneficial behavioral disease outcomes. However, a dose–response study may be needed to confirm the ideal ApoA1 treatment schedule. Potentially, repeated ApoA1 injections during disease progression may be more beneficial. Capillary permeability detection via intravenous injection of the fluorescent EB dye was reported in a number of our publications (Garbuzova-Davis et al., 2007b, 2017, 2018, 2019, 2021) and provided useful information for the evaluation of microvascular status in the spinal cords of mice.

Other important findings of the present study included significant reductions of astrogliosis and microgliosis along with re-establishment of perivascular astrocytes surrounding cervical/lumbar spinal cord microvessels of ALS animals of both genders treated with ApoA1. ApoA1 is a multifunctional protein, exerting numerous positive outcomes in the treatment of several diseases (Smith, 2010; Richart et al., 2020, p. 2; Cochran et al., 2021; Bhale and Venkataraman, 2022; Xu et al., 2022). Moreover, ApoA1 provided anti-inflammatory effects through maintenance of vascular ECs (Yan et al., 2006; Li et al., 2008; Puranik et al., 2008; Pan et al., 2016; Svensson et al., 2017). Thus, decreased morphological signs of astrocyte cell reactivity and microglial cell activation in the spinal cords might provide ApoA1’s anti-inflammatory effect by possibly mitigating CNS vascular damage in male and female ALS mice. However, humoral pro- and anti-inflammatory cytokine profiles in ALS mice of both genders are needed to confirm our suggestion.

Finally, analysis of administered human ApoA1 into ALS mice of both genders showed protein distribution within numerous microvessels and cervical/lumbar spinal cord motor neurons of treated animals. Also, ApoA1 was detected in capillary endothelium or outside of microvessels. Although the specific mechanisms by which ApoA1 enters ECs *in vivo* are not clearly understood, this finding was supported by our *in vitro* study (Garbuzova-Davis et al., 2022) showing ApoA1 incorporation into ECs via the PI3K/Akt downstream signaling pathway. However, ApoA1’s appearance in motor neurons in the spinal cords of ALS-treated mice of both genders requires further discussion. It is well known that the neurovascular unit (NVU) plays an essential role in proper CNS function [reviewed in Lecrux and Hamel (2011); Yu et al. (2020), and Zhou et al. (2023)]. The NVU, composed of microvascular cells, including ECs, astrocytes, and neurons, regulates the delivery of oxygen and/or nutrients to the CNS and expels metabolic byproducts. Disruption of any NVU components may contribute to the pathogenesis of neurodegenerative diseases. Particularly, NVU dysregulation in ALS (Garbuzova-Davis et al., 2011) is related to the blood–CNS barrier impairment, which leads to motor neuron degeneration. This widely accepted pathogenic mechanism of ALS is based on EC impairment, degeneration of tight junctions, and reduction in astrocytic end-feet surrounding capillaries. In the current study, we have shown that ApoA1 treatment re-established perivascular astrocyte capillary coverage and likely maintained suitable EC function in the spinal cord in mice of both genders, resulting in NVU restoration. Additional supporting evidence was the detection of numerous potential ApoA1 particles within healthy motor neurons in the lumbar spinal cord after ApoA1 treatment. The present data reinforce our previous results supporting ApoA1 as a potential noncellular therapeutic for the repair of the damaged blood-CNS barrier in ALS (Manora et al., 2024). However, long-term effects of ApoA1, such as determining the lifespan of ALS mice in both genders, should be evaluated prior to establishing the feasibility of ApoA1 as a treatment for ALS.

In conclusion, our study provided new evidence supporting the benefits of systemically administered ApoA1 in ALS mice of both genders at the early symptomatic disease stage, likely due to restorative effects on damaged microvessels, toward repair of the blood–spinal cord barrier. Results of our preclinical study suggest ApoA1 as a potential protein therapeutic for re-establishing vascular integrity in ALS. The provided figures directly support our findings, coupled with the statistical analysis accompanying our data, to further strengthen the conclusions. Moreover, the study has translational value since the treatment was initiated at the symptomatic disease stage of ALS mice. Thus, this innovative strategy may engender clinical trials on the road to effectively treating ALS patients.

## Data Availability

The raw data supporting the conclusions of this article will be made available by the authors, without undue reservation.
